# Intracardiac Teratoma in an Infant: Report of a New Case and Literature Review

**DOI:** 10.1155/2018/6805234

**Published:** 2018-06-10

**Authors:** Mustapha Azzakhmam, Amine Kessab, Abderrahmane Malihy, Lamiae Rouas, Najat Lamalmi

**Affiliations:** ^1^Department of Pathology, Military Hospital of Rabat, Rabat, Morocco; ^2^Department of Pathology, Pediatric Hospital of Rabat, Rabat, Morocco

## Abstract

Primitive intracardiac tumours are rare, especially in childhood, and are often discovered on autopsy. The intracardiac teratoma is the rarest intracardiac tumours of childhood. Herein, we report the case of an 11-month-old infant, which featured recurrent bronchoalveolitis since the age of 3 months, with a thoracic deformation. Physical examination did found discrete respiratory distress signs. Chest radiography showed large mediastinal enlargement. The computed tomography showed a solid cystic-cloisonned mass with fat and central calcification highly suggestive of an intracardiac teratoma. A radical surgical excision was made and the histological examination found a well circumscribed tumour containing elements of the three germ layers confirming the diagnosis of mature well-differentiated teratoma, with no need of immunohistochemical support.

## 1. Introduction

Primary cardiac tumours are extremely rare: their impact would be of the order of 1.7 to 2.8 per 1000 autopsies in all ages according to studies published in the literature [1, 2]. This impact is even lower among the pediatric population and reported to be 2.5 per 1000 children. Cardiac metastasis of other primary tumours would be 20 to 30 times more frequent.

The first description, which dates back to 1959, was made on autopsy. BARNES made the first clinical diagnosis in 1934 [3]. In more than 75% of cases, intracardiac tumours are benign and are represented especially by myxoma (50%) and rhabdomyoma (20%). Cardiac primitive malignancies are especially dominated by sarcomas (20%).

The primitive cardiac teratoma is extremely rare; the cases reported in the literature are limited to a dozen cases and they are approximatively 1–5% of all pediatric neoplasms [4, 5]. Intracardiac location is even more exceptional [6, 7].

## 2. Case Report

The mother was 29 years old and had no history of medical problems nor antecedents of congenital anomalies or tumours. The pregnancy was well followed up.

We report the case of an 11-month-old male infant who presented since the age of three months recurrent episodes of bronchoalveolitis. Moreover, since the age of 5 months, he presented a thoracic deformation. The clinical examination revealed a thoracic deformation with discrete respiratory distress. The patient was well colored and well hydrated.

**Figure 1:**
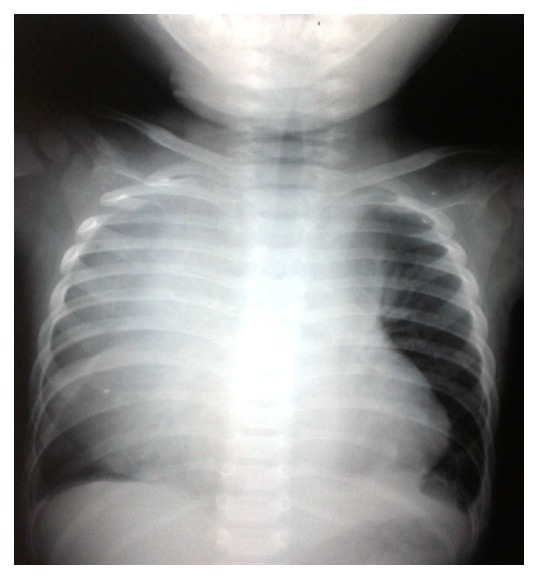
Chest radiography showing large homogeneous mediastinal opacity with mediastinal enlargement.

A chest radiography was performed and showed a large homogeneous mediastinal opacity with mediastinal enlargement (Figure 1).

**Figure 2:**
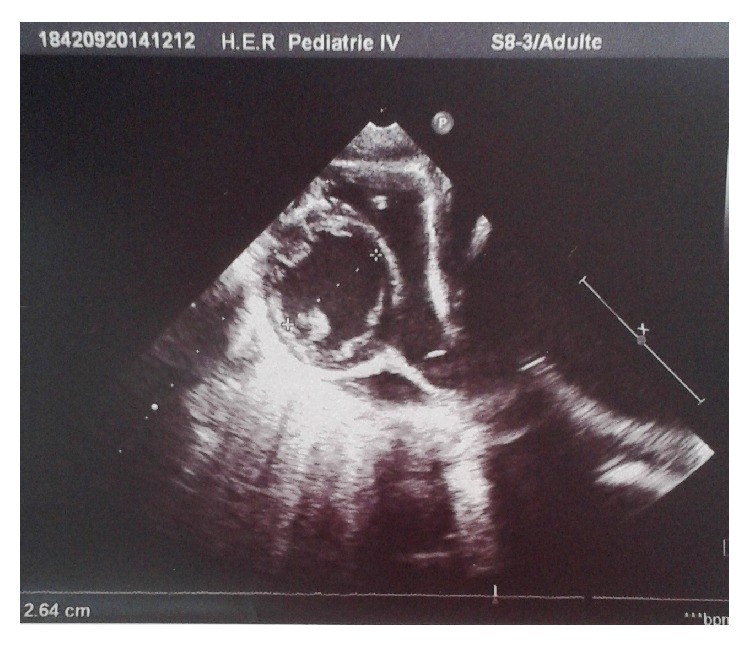
Echocardiography showing a 15 cm heart mass compressing the two atria.

Echocardiography showed a 15 cm heart mass compressing the two atria (Figure 2).

The thoracic echography showed a large process of the upper mediastinum, on the right side of the pericardium, measuring 116 × 79 × 76 mm with a double component: a major cystic-cloisonned component and a fleshy well vascularised component. This process was well circumscribed concording with a teratoma.

**Figure 3:**
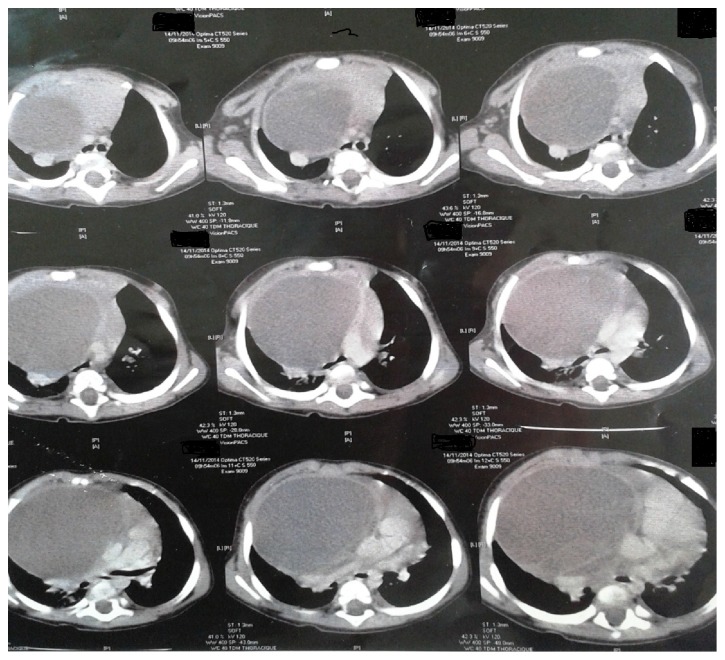
CT scan showing aspects of mediastinal teratoma: a combined cystic-solid tumour with walls of variable thickness.

The CT scan showed aspects of mediastinal teratoma consisting of a combined cystic-solid tumour with walls of variable thickness. The combination of fluid, soft tissue, a fat-fluid level produced by high lipid content in the cyst fluid as seen in our case is a rare but diagnostic sign (Figure 3).

It was a cystic-cloisonned well circumscribed tumour, showing thick cloisons; this aspect was highly suggestive of a teratoma more than a cystic lymphangioma. This tumour was repressing the heart and the thymus to the left side, the pulmonary veins to the back, and the aorta and the pulmonary artery to the back (Figure 3).

Biological examination showed a deep anaemia with 11 g/l of haemoglobin and no elevated levels of alpha protein. The infant was referred to surgery for process excision.

The surgical exploration found an anterior-well-encapsulated mediastinal mass with double component, which was in close contact with the superior vena cava outside and the right atrium at the bottom and with the ascending aorta behind. A careful dissection of the tumour, which was in intracardiac localisation, was performed because of its intimate connections with superior vena cava, and with the right atrium, and particularly with the ascendant aorta. A total excision of the tumour and a focal pericardiectomy were performed.

## 3. Gross Examination

**Figure 4:**
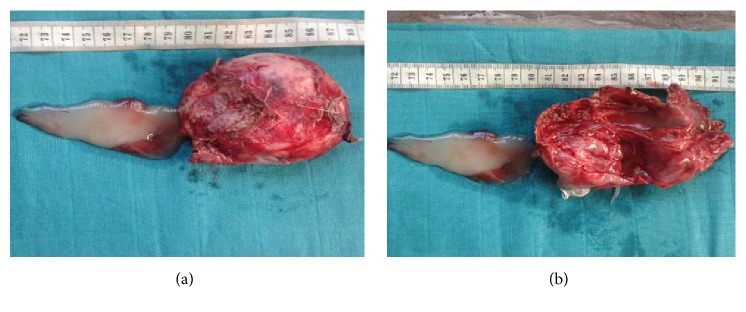
Gross examination: the opened mass showed a double component: a cystic and a fleshy one, with an outcome of a mucous material at the opening ((a)-(b)).

It was a 110 g weight mass and measuring 10 × 10 × 3 cm. At the time of opening the mass, there has been an outcome of a mucous material. Moreover, the mass was well encapsulated. In the time of examination of the open mass, we found a double component; a cystic and a fleshy component (Figure 4).

## 4. Histological Examination

**Figure 5:**
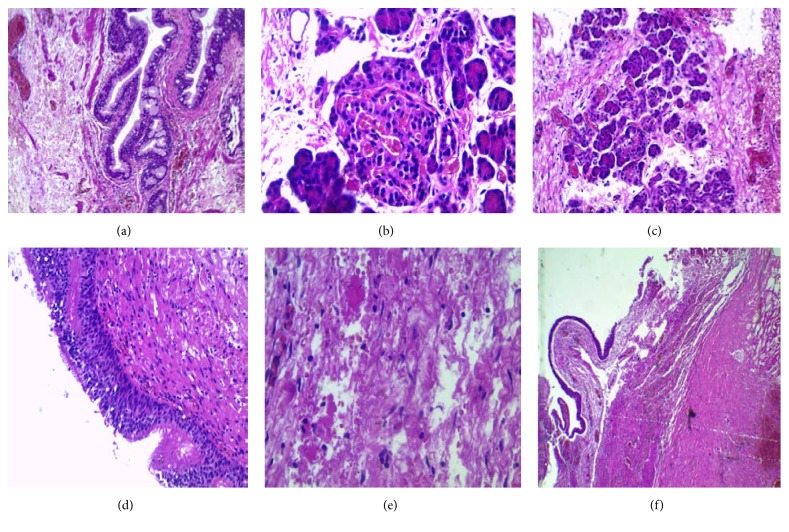
Histological examination of the resected mass [HE.GX20]: (a) regular glandular gastric mucosa into sclerotic and muscular tissue. (b) Hepatic regular tissue. (c) Pancreatic tissue showing pancreatic islets of Langerhans. (d) Columnar pseudo-stratified-ciliated respiratory epithelioma. (e) Mature nervous tissue. (f) Pericardium with strips of Malpighian epithelioma.

Multiple samples of the process were taken and showed a multitissue proliferating process. It includes cystic cavities lined by a ciliated respiratory epithelioma, a gastric mucosa surrounded by soft muscle bundles, a regular Malpighian epithelioma, a mature nervous tissue, pancreatic tissue, and hepatic tissue (Figure 5).

A sample was separately received, matched with a fibrous tissue with mononucleated inflammatory cells and showing focally, a regular Malpighian epithelioma.

The histological study of the following resected tumour supported the final diagnosis of benign teratoma.

## 5. Discussion

Intracardiac teratomas are very rare tumours, little encountered in clinical practice, and confined practically to the pediatric population [4].

Morphologically, these tumours can show complex and varied aspects due to the histological variety of their constituents, and they are derived from one or several layers of germ cells. These findings explain the diagnosis difficulties faced sometimes by the pathologist, when the tumour is not well differentiated or immature. In our case, the tumour consisted of well-differentiated mature teratomatous elements allowing a morphology based diagnosis with no need for immunohistochemical studies.

Clinically, the symptomatology can be variable. It depends largely on the tumour location: the patient may be asymptomatic if the tumour is located far from the outflow tract, as in the case of the rhabdomyomas. When the tumour is located within these flow paths, it can generate their obstruction and therefore produce moderate to severe hemodynamic disorders. These signs can be detected early by echocardiography, even in the prenatal period.

**Figure 6:**
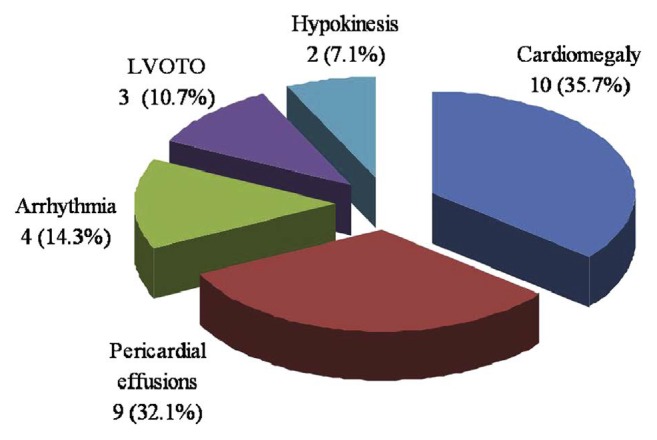
Echocardiographic findings of fetal primary cardiac tumours. 10 LVOTO = left ventricular outflow tract obstruction.

Echocardiography plays a crucial role in the diagnosis of intracardiac tumours, particularly in the prenatal diagnosis of neoplasms. Furthermore, it plays a role in the guidance of in utero-pericardiocentesis. By far, cardiomegaly and pericardial effusions are the most common echocardiographic findings in fetoneonatal primary cardiac tumours (Figure 6) [10].

The most common location reported in the literature is the atrioventricular surface adjacent to the tricuspid valve, and the most clinical reported presentation is represented by congestive heart failure [6, 7, 10, 11]. In our case, clinical signs were nonspecific and were limited to discrete respiratory signs.

**Figure 7:**
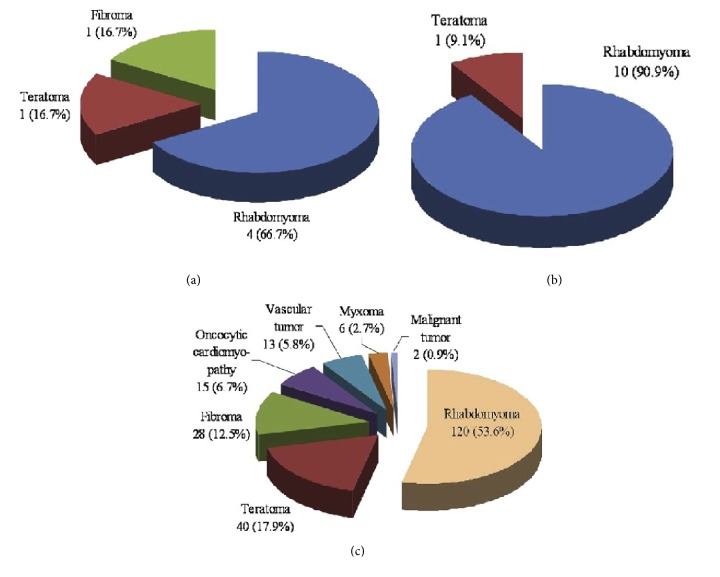
Incidences of primary cardiac tumours in (a) fetal [8], (b) fetal [9], and (c) fetoneonatal groups [9].

Intracardiac teratomas represent a small percentage of primitive intracardiac tumours in children. In the literature they are limited to a dozen reported cases [9, 12, 13]. Teratomas represent 16,7% of all primary fetal cardiac tumours, versus 66,75% for rhabdomyomas in the large series of NIEWADOMOSKA concerning 23 cases [8]. In the study of ISAACS [9], the incidence of teratomas was 9,1% versus 90,9% for rhabdomyomas in fetal groups. No significant differences were seen between the incidence of fetal primary cardiac tumours and that of infantile groups (0,11% versus 0.08%) according to ISAAC, and incidence of teratomas in the fetoneonatal groups was up to 17,9% [9] (Figure 7).

In addition, some cases reported were not real teratomas, as the case reported by A. De Chatel (1933) [12] and the case of K. Niewiadomska-Jarosik (1950) [8] but rather a pseudo cyst of epithelial inclusions. Moreover, malignant teratomas are distinguished apart [14].

The pathogeny of primitive cardiac teratomas is not well known, and the theory of migration of totipotent germ cells is the most accepted to explain their origins. According to this theory, the primitive germ cells can, during their migration, get lost or stopped before completing their migration and be included to the atrioventricular canal during embryonic development and potentially develop later into teratoma [15, 16]. This theory also explains the tendency of these teratomas to arise from the atrioventricular or interatrial septum [1, 2, 17].

Histologically, the benign teratomas are often cystic, multicystic, or solid-multicystic and show elements derived from all three germ layers (endoderm, ectoderm, and mesoderm) including bone, cartilage, smooth muscle, liver, pancreas, and neural tissue [16]. The cysts can be lined by a pseudostratified ciliated respiratory epithelium, columnar epithelium, or squamous epithelium. These aspects define the mature well-differentiated teratomas, as the case we report. There were only a few reports regarding malignant germ cell tumours in young children [13]. Furthermore, other malignant intracardiac tumours are even rare and have been rarely reported in the literature, such as the case of primitive pericardial undifferentiated sarcoma of a 9-year-old boy, reported by LAZARUS [18].

The differential diagnosis of intracardiac teratomas is represented by other most frequent cardiac tumours, especially the epithelial cyst of the myocardium. Other cardiac tumours may also have a cystic presentation particularly: the mesothelioma of the atrioventricular node, the myxoma, and vascular tumours such as haemangioma and sarcoma [14].

The only therapeutic management is complete surgical excision, because incomplete resection may be the cause of recurrence. The outcome reported in the literature remains poor despite the benign character, and death occurs following the evolutionary complications of these tumours [19]. Apart from the histological type, evolution also depends on the size, the number, and location of the tumours. Serious complications that may arise are disorders of cardiac rhythm, explained by frequent location within the nodal tissue. In a large series of 23 intracardiac tumour cases diagnosed prenatally, five newborns underwent a partial or complete tumour excision, and 11 have died including three at least by congestive heart failure [8]. In our case, during a follow-up extending beyond 35 months, there has been no clinical or echocardiographic evidence of any residual cardiac disease, and the patient is doing well.

In conclusion, even rare, intracardiac teratoma must be kept in mind when considering an intracardiac tumour by clinicians and especially by pathologists. A large sampling of the resected specimens must be the rule and eventually supported after microscopic examination, by an immunohistochemical study in complex cases.
